# Epidemiological models for predicting Ross River virus in Australia: A systematic review

**DOI:** 10.1371/journal.pntd.0008621

**Published:** 2020-09-24

**Authors:** Wei Qian, Elvina Viennet, Kathryn Glass, David Harley

**Affiliations:** 1 Mater Research Institute‐University of Queensland (MRI‐UQ), Brisbane, Queensland, Australia; 2 Research and Development, Australian Red Cross Lifeblood, Brisbane, Queensland, Australia; 3 Institute for Health and Biomedical Innovation, School of Biomedical Sciences, Queensland University of Technology (QUT), Queensland, Australia; 4 Research School of Population Health, Australian National University, Acton, Australian Capital Territory, Australia; Oregon State University College of Veterinary Medicine, UNITED STATES

## Abstract

Ross River virus (RRV) is the most common and widespread arbovirus in Australia. Epidemiological models of RRV increase understanding of RRV transmission and help provide early warning of outbreaks to reduce incidence. However, RRV predictive models have not been systematically reviewed, analysed, and compared. The hypothesis of this systematic review was that summarising the epidemiological models applied to predict RRV disease and analysing model performance could elucidate drivers of RRV incidence and transmission patterns. We performed a systematic literature search in PubMed, EMBASE, Web of Science, Cochrane Library, and Scopus for studies of RRV using population-based data, incorporating at least one epidemiological model and analysing the association between exposures and RRV disease. Forty-three articles, all of high or medium quality, were included. Twenty-two (51.2%) used generalised linear models and 11 (25.6%) used time-series models. Climate and weather data were used in 27 (62.8%) and mosquito abundance or related data were used in 14 (32.6%) articles as model covariates. A total of 140 models were included across the articles. Rainfall (69 models, 49.3%), temperature (66, 47.1%) and tide height (45, 32.1%) were the three most commonly used exposures. Ten (23.3%) studies published data related to model performance. This review summarises current knowledge of RRV modelling and reveals a research gap in comparing predictive methods. To improve predictive accuracy, new methods for forecasting, such as non-linear mixed models and machine learning approaches, warrant investigation.

## Author summary

As the most common human arbovirus infection in Australia, Ross River virus exerts a significant public health and economic burden on the population. Because the virus is transmitted by mosquitoes, incidence is influenced by climate, environment, and socio-economic factors. Using epidemiological models to predict incidence or outbreaks of RRV fully utilises these data to inform decision-making. In this systematic review, we summarised models and their predictive performance, and highlighted significant exposures in order to increase understanding of transmission.

## Introduction

Ross River virus, a mosquito-transmitted *Alphavirus*, is the most common arboviral infection of humans in Australia [1, 2] and often results in a characteristic syndrome, including constitutional effects, rash, and rheumatic manifestations [1, 3]. A total of 123,875 cases of RRV infection were reported from 1993 to 2019 in Australia, of which nearly half (48.8%) were from Queensland. [4]

Ross River virus transmission is primarily influenced by mosquito abundance, reservoir host populations, and climatic, environmental (e.g. rainfall, temperature, tides, river flow, vegetation cover) and socio-economic factors (e.g. urban development, housing infrastructure) [1, 5–9]. Models of RRV using these exposures can improve knowledge of RRV transmission or be used to give early warning of outbreaks, thus aiding disease prevention and control. However, the relationships between exposures and RRV incidence are complex. For instance, climate can influence vector abundance, host populations and the behaviour of vectors and hosts, and climate and weather are influenced by human behaviour (e.g. global warming, heat island effects, effects of large dams) and geographical factors like altitude [10, 11]. Therefore, exposures do not have a simple correlation with disease incidence, which increases the difficulty of forecasting.

Generalised linear regression and time-series models are widely used for infectious disease prediction [12]. Linear regression models are straightforward, but often inadequate for prediction in complex systems. Time-series models are especially suitable for analysing data containing autocorrelation and which shows periodic fluctuations [13, 14].

Three reviews on exposures or predictive models of RRV have been published, however all concentrated on a description of exposures and their relationships with disease, with less attention to models and their performance, and none were systematic reviews. A review by Tong et al. (2008) [8] included more than 15 articles on predictors of RRV transmission. Analytical methods were listed, and the detailed research process and results were described to elucidate the association between climatic, social and environmental factors and RRV disease. Yu et al. (2014) [15] identified research on the impact of climate change on RRV disease. All models applied in these studies were listed, but the characteristics of the models were not discussed. Another review by Jacups et al. (2008) [9] described the vectors and vertebrate reservoirs of RRV, the possible impact of climate change on incidence, and summarised the models and the climatic factors applied in 15 studies. RRV models were discussed in this study, but the focus was on the influence of covariates and the geographical size of the study areas on the model accuracies. However, these three reviews neither provide a detailed profile of the models nor quantification of their performance.

In this review, the research hypothesis is that a detailed summary of all available primary modelling research for RRV enables an evaluation of the effectiveness of the models in forecasting disease and improving knowledge of exposures and transmission cycle. We aim to describe modelling approaches applied in RRV disease prediction in Australia, the performance of these models, the variables used and the models’ performance in prediction.

## Methods

This systematic review followed the Preferred Reporting Items for Systematic Reviews and Meta-Analyses (PRISMA) guidelines [16]. The PRISMA Checklist is available in S1 Table. The proposal for this study was completed before data extraction (S1 Text).

### Literature search strategy

We performed a structured literature search using PubMed, EMBASE, Web of Science, Cochrane Library, and Scopus for articles published between January 1, 1980 and January 21, 2020 with search terms encompassing pathogen (i.e. “Ross River virus”), methods (i.e. “model” OR”forecast”), and exposures (i.e. “impact factor” OR “predictor” OR “association”). Articles not relevant to our aims were excluded (i.e. “gene” OR “protein” OR “transfusion”). These search terms efficiently excluded irrelevant records. Studies on genes or proteins were mainly laboratory-based and not relevant for epidemiological risk prediction. Studies on transfusion-related RRV transmission were excluded because such transmission is infrequent and can be ignored. In addition, findings could not feasibly be integrated with studies of mosquito-transmitted disease. Search terms are provided in S2 Text.

### Inclusion and exclusion criteria

Studies of RRV using population-based data, incorporating at least one epidemiological model and analysing the association between an exposure or exposures and RRV incidence or outbreaks, located in Australia, in English and with full-text available were included in this review. Review articles, meeting abstracts, letters, books, reports and comments were excluded. Studies on RRV virology, vaccines, and animal models were also excluded. Articles describing models of RRV vectors or non-human reservoir hosts without human epidemiological data were excluded, as were studies involving transmission dynamic modelling. The records were first screened by titles and abstracts, then the full texts were reviewed before a final decision on inclusion. Study inclusion was conducted by one author (WQ), and in cases of uncertainty, all four authors reached a decision after discussion.

For included records, title, author, publication year, research area and period, predictors and format of predicted outcome, modelling method, significant results, prediction performance, model evaluation and model validation were extracted.

### Methodological quality assessment and data extraction

Included studies were assessed according to recently published criteria for observational studies [17–20]. Each criterion was scored 2, 1 or 0 if the studies fully, partly or barely met the criterion (S2 Table). The statement of funding and conflict of interest were each scored 1 if they were stated clearly. The studies were classified into three groups depending on total scores: high (19–24), medium (13–18), and low quality (<13). Related study registrations were searched to evaluate publication bias. Data extraction and quality assessment were conducted by one author (WQ) and discussed by all authors where there were uncertainties.

All the authors participated in the entire review process, discussed the main decisions and reached agreement on study selection, data extraction and study assessment together.

Study characteristics and model performance were tabulated. Exposures applied in these models were summarised and their association with RRV listed.

**Fig 1:**
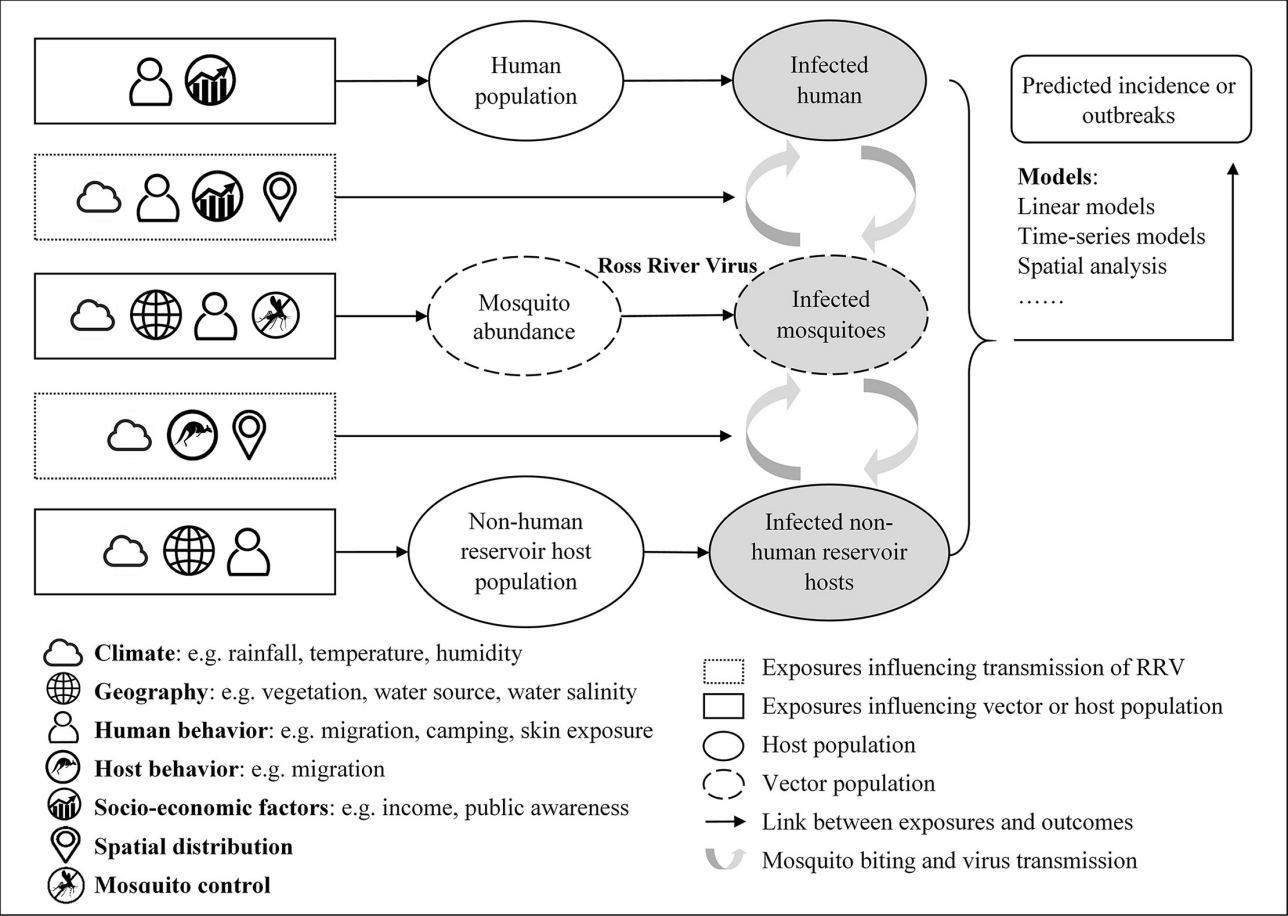
Key elements of RRV prediction models.

The transmission cycle of RRV and key exposures influencing RRV infection are illustrated in Fig 1.

## Results

**Fig 2:**
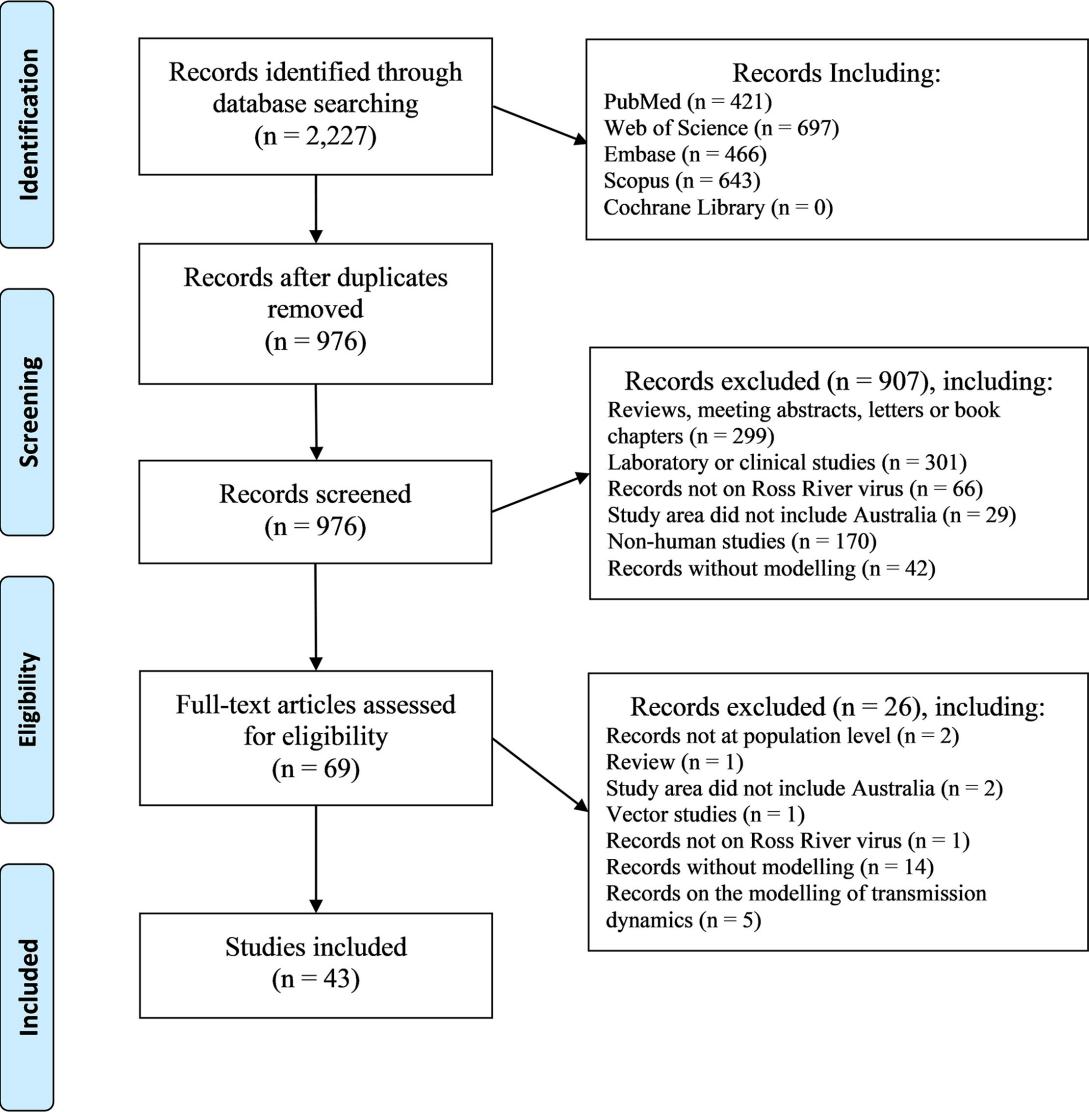
Flow chart of study selection process.

After duplicates were removed from 2,227 searched records, we screened 976 papers; after exclusion criteria were applied, 43 records remained (Fig 2) [21–63]. All studies were published in the last 20 years, and 19 (44.2%) during the past decade. Nearly half the studies were conducted in Queensland (20, 46.5%), while five (11.6%) were in Western Australia.

The quality scores for the studies are listed in Table 1. Detailed scores are provided in S3 Table. All studies attained high (33, 76.7%) or medium quality scores (10, 23.3%). There were two articles published without significant results. No systematic review registration related to RRV modelling was found.

**Table 1:** Score for quality assessment criteria.

Assessment criterion	Number (%) of articles scoring 2*	Number (%) of articles scoring 1	Number (%) of articles scoring 0
1. Clarity of aims and objectives	43 (100.0)	0 (0.0)	0 (0.0)
2. Definition of study geographical area	40 (93.0)	3 (7.0)	0 (0.0)
3. Data source of RRV clearly described	42 (97.7)	1 (2.3)	0 (0.0)
4. Data source of covariates clearly described	43 (100.0)	0 (0.0)	0 (0.0)
5. Data quality considered	22 (51.2)	5 (11.6)	16 (37.2)
6. Model structure clearly described and appropriate for the research question	41 (95.3)	2 (4.7)	0 (0.0)
7. Modelling methods appropriate for the research question	42 (97.7)	1 (2.3)	0 (0.0)
8. Model evaluation	25 (58.1)	3 (7.0)	15 (34.9)
9. Model validation	14 (32.6)	1 (2.3)	28 (65.1)
10. Results clearly and completely presented	36 (83.7)	7 (16.3)	0 (0.0)
11. Results appropriately interpreted and discussed in context	40 (93.0)	3 (7.0)	0 (0.0)
12. Funding statement	-	34 (79.1)	9 (20.9)
13. Conflict of interest statement	-	17 (39.5)	26 (60.5)

*The assessment of Funding statement and Conflict of interest statement was scored 1 or 0.

Models were grouped into three categories: generalised linear models (22 studies, 51.2% of 43 studies); time-series models (11, 25.6%); other models including Cumulative Sum based (CUSUM-based) methods (3, 7.0%), spatial or temporal analysis (5, 11.6%), Classification and Regression Tree (CART) (2, 4.7%), Maxent model, Hurdle model, Besag, York, and Mollié (BYM) model and generalised additive model (1 each, 2.3%) (Tables 2–4). In some studies, different types of models, or models with different covariates were compared. A total of 140 models were built in these 43 studies.

**Table 2:** Characteristics of the models predicting RRV incidence rates.

Study (first author, year)	Research area and period	Model*	Covariates (significant predictors in bold)**
Ryan, 1999	Maroochy Shire, 1991–1996	LIRM	**Mosq**
Muhar, 2000	Brisbane, 1991–1996	LIRM	**Vegetation** types obtained by principal component factor analysis
Tong, 2001	Cairns, 1985–1996	ARIMA	**Rain**, Temp, **Humd**, Tide
Done, 2002	Queensland, 1991–1997	Second-order auto-regression model	Stratospheric Quasi-Biennial Oscillation index, the annual, semi-annual and quasi-biennial cycles **(significant predictors not presented)**
Tong, 2002(1)	Queensland, 1985–1996	ARIMA	**Rain**, Temp, **Tide**, adjustment for auto-regression, moving average, seasonality, and year
Tong, 2002(2)	Queensland, 1985–1996	PTSRM	**Rain**, **Temp**, **Humd**, **Tide**, adjustment for seasonality, case notification time and human population (offset variable)
Hu, 2004	Brisbane, 1985–2001	SARIMA	**Rain**, Temp, Humd, Tide, **auto-regression**, **moving average**, **seasonal auto-regression**, **seasonal moving average**
Tong, 2004	Townsville, 1985–1996	SARIMA	**Rain**, Temp, Humd, **Tide**
Gatton, 2004	Queensland, 1991–2001	Spatial analysis	**Spatial autocorrelation**
Tong, 2005	Brisbane, 1998–2001	PTSRM	**Mosq**, **Rain**, **Tide**, SOI, adjustment for the autocorrelation and seasonality
Hu, 2006(1)	Brisbane, 1998–2001	PTSRM and CART	**Mosq**, adjustment for overdispersion, Max Temp, autocorrelation, and seasonality
Hu, 2006(2)	Brisbane, 1998–2001	PDL and SARIMA	**Mosq**, Rain, Tide, SOI, **seasonal auto-regression** for SARIMA model, adjustment for seasonality and autocorrelation
Ryan, 2006	Redland Shire, 1991–2001	Spatial analysis	Mosq **(significant predictors not presented)**
Hu, 2007	Brisbane, 2001	NBRM	**SOI**, human population, overseas visitors, indigenous population, labor workers, **educational level**, family income, **vegetation**, seasonality
Jardine, 2008	Southwestern Australia, 1988–2006	NBRM and Besag, York, and Mollié (BYM) model	Land salinity, waterlogging, human population (offset variable)
Bi, 2009	The Riverland region of South Australia, 1992–2004	Poisson regression model	**Rain**, **Temp**, **Humd**, **SOI**, **River Murray flow**, **historical RRV cases**, adjustment for autocorrelation, seasonality and lagged effect
Hu, 2010 (1)	Queensland, 1999–2001	Bayesian spatio-temporal conditional auto-regressive model	**Rain**, **Temp**, SEIFA, **spatial variation**, **LGA-specific temporal trends**, a seasonally oscillating temporal random effect, human population (offset variable)
Hu, 2010 (2)	Brisbane, 1998–2001	CART	**Mosq**
Pelecanos, 2011	Queensland, 1995–2007	Spatial and temporal analysis	**Spatial autocorrelation**
Vally, 2012	Three regions in Western Australia, 1995–1996	Poisson linear regression model	**Distance from the waterway**, human population (offset variable)
Yu, 2014	Queensland, 2001–2011	Spatial and temporal analysis	**Spatial autocorrelation**
Koolhof, 2017	Five sites in Western Australia, 1991–2014	LIRM	**Rain**, **Temp**, **Tide**
Stratton, 2017	Australia, 1993–2015	Poisson linear regression model	Rain, Temp or TSI, trend, seasonality, **intra-annual periodicity**, inter-annual periodicity, time-lag

* LIRM = Linear Regression Model; ARIMA = Auto-Regressive Integrated Moving Average model; SARIMA = Seasonal Auto-Regressive Integrated Moving Average model; PTSRM = Poisson Time Series Regression Model; CART = Classification and Regression Tree; PDL = Polynomial Distributed Lag model; NBRM = Negative Binomial Regression Model.

**Mosq = Mosquito abundance or Mosquito related data; Rain = Rainfall; Temp = Temperature; Tide = Tide Height or High Tidal level; Humd = Humidity or Relative Humidity; SOI = Southern Oscillation Index; SEIFA = Socio-Economic Index for Areas; TSI = Temperature Suitability Index.

**Table 3:** Characteristics of the models predicting RRV outbreaks.

Study (first author, year)	Research area and period	Model*	Covariates (significant predictors in bold)**
Maelzer, 1999	New South Wales and Victoria, 1928–1998	LORM	**SOI**
Woodruff, 2002	Two regions in southeastern Australia, 1991–1999	LORM	**Rain**, **Temp**, **Humd**, Evap, **VP**, SOI, **SST**, adjustment for irrigation method
Gatton, 2004	Queensland, 1991–2001	Temporal analysis	**Seasonality**
Gatton, 2005	Queensland, 1991–2001	LORM	Rain, Temp
Woodruff, 2006	Southwest Western Australia, 1991–1999	LORM	**Mosq**, **Rain**, Temp, **Tide**, Evap, SOI, SST, VP
Watkins, 2008	Western Australia, 1991–2004	EARS and NBC	Historical RRV cases **(covariates not quantified)**
Pelecanos, 2010	Queensland, 1991–2007	EARS, NBC, HLM, POD and temporal analysis	Historical RRV cases **(covariates not quantified)**
Sparks, 2010	New South Wales, 1995–2006	An adaptive CUSUM plan	Historical RRV cases, day of the week, school holiday, seasonal and transitional influences **(covariates not quantified)**
Jacups, 2011	Northern Territory, 1991–2007	LORM	**Rain**, **Temp**, **Humd**, **Tide** (only applied for coastal areas)
Koolhof, 2017	Five sites in Western Australia, 1991–2014	Hurdle model	**Rain**, **Temp**, **Tide**
Walsh, 2018	Australia, 1996–2016	Maxent model	Rain, Temp, proximity to each surface water type, **proximity to controlled water reservoirs**, **water-soil balance**, **hydrological flow accumulation**, **altitude**, **MGVF**, human migration, **ecological niches of wildlife hosts**
Tall, 2019	Inland New South Wales, 1991–2013	GEE	**Flood events**

* LORM = Logistic Regression Model; EARS = Early Aberration Reporting System C1, C2 and C3 algorithms; CUSUM = Cumulative Sum; NBC = Negative Binomial Cusum method; HLM = Historical Limits Method; POD = Poisson Outbreak Detection method; GEE = Generalised Estimating Equations.

**Mosq = Mosquito abundance or Mosquito related data; Rain = Rainfall; Temp = Temperature; Tide = Tide Height or High Tidal level; Humd = Humidity or Relative Humidity; Evap = Evaporation; VP = Vapor Pressure; SOI = Southern Oscillation Index; SST = Sea Surface Temperature; MGVF = Maximum Green Vegetation Fraction.

**Table 4:** Characteristics of the models predicting RRV cases.

Study (first author, year)	Research area and period	Model*	Covariates (significant predictors in bold)**
Jacups, 2008	Darwin, 1991–2006	Poisson linear regression model	**Mosq**, **Rain**, **Temp**, Humd, Tide
Barton, 2009	The Gippsland Lakes region of eastern Victoria, 1991–2001	LIRM	**Mosq**, **Rain**, **Temp**
Williams, 2009	The River Murray Valley of South Australia, 1999–2006	LIRM	**Mosq**, **Rain**, **Temp**, **river height**, human population
Werner, 2012	Southeastern Tasmania, 1993–2009	NBRM	**Rain**, **Temp**, Tide
Ng, 2014	Four regions in New South Wales, 1991–2004	PDL	**Mosq**, **Rain**, **Temp**, **Humd**, **Tide**, **Evap**, **SST**, **NDVI**, **water sources**, distance to coast, elevation, **ARIA**, **macropod population**, human population (offset variable)
Rohart, 2016	Australia, 2009–2013	High-dimensional LIRM	Google Trends data **(covariates not quantified)**
Cutcher, 2017	The Mildura Local Government Area of Victoria, 2000–2015	NBRM	**Mosq**, **Rain**, Temp, Humd, **VP**, **SOI**, La Niña events, **SST**, sea level, **river flow**
Flies, 2018	South Australia, 1992–2012	Generalised additive model	Mosq, Rain, **Temp**, Humd, **distance to coast**, **distance to Murray River**, NDVI, **elevation**, **IRSD,** Caravan parks, **global human settlement "urban-ness" score**, **non-human reservoirs**, **expected RRV cases**
Walker, 2018	The Peel region of southwest Western Australia, 2003–2014	NBRM	**Mosq**, **presence of RRV isolates**, **season**
Koolhof, 2019	The eleven Local Government Areas in Victoria, 2005–2018	NBRM	**Rain**, **Temp**, **Humd**, **Tide**, **VP**, **sea level pressure**, **Evap**, SOI, **SST**, **river flow**

* LORM = Logistic Regression Model; LIRM = Linear Regression Model; PDL = Polynomial Distributed Lag model; NBRM = Negative Binomial Regression Model.

**Mosq = Mosquito abundance or Mosquito related data; Rain = Rainfall; Temp = Temperature; Tide = Tide Height or High Tidal level; Humd = Humidity or Relative Humidity; Evap = Evaporation; VP = Vapor Pressure; SST = Sea Surface Temperature; SOI = Southern Oscillation Index; NDVI = Normalized Difference Vegetation Index; ARIA = Accessibility/Remoteness Index of Australia; IRSD = Index of Relative Socio-economic Disadvantage.

Fourteen articles (32.6% of 43 articles) used mosquito abundance or related data as model covariates. Climate and weather data were used by 27 of the 43 studies (62.8%). Other exposures such as river flow, distance to surface water sources, historical RRV cases and host population were also used (Table 5). Rainfall (applied in 69 models, 49.3% of 140 models), temperature (66, 47.1%) and tide height (45, 32.1%) were the three most commonly used risk factors for predicting RRV infection. The full list of 65 exposures in 7 categories, their associations with RRV and time-lags are provided in S4 Table.

**Table 5:** Top 15 significant exposures applied, and the directions of their associations with RRV.

Exposures*	N_sa_ / N_a_**	N_sm_ / N_m_**	Association with RRV (Positive / Negative) ***
Rainfall	20 / 25	45 / 69	47 / 14
Temperature or Temperature Suitability Index	14 / 23	35 / 66	31 / 15
Tidal height or high tidal level	10 / 15	19 / 45	17 / 6
Mosquitoes, with top three: *Culex annulirostris*, *Aedes camptorhynchus* and *Culex australicus*	12 / 14	23 / 42	43 / 2
Humidity or relative humidity	7 / 12	12 / 37	5 / 10
Southern Oscillation Index	4 / 9	6 / 15	5 / 1
Sea Surface Temperature	4 / 5	6 / 22	4 / 6
River flow or river height	5 / 5	5 / 19	5 / 2
Vapor pressure	3 / 4	5 / 18	5 / 0
Distance to each surface water type	3 / 4	3 / 9	0 / 4
Evaporation	2 / 4	7 / 21	8 / 1
Seasonality / Season	2 / 4	7 / 9	7 / 0
Historical RRV cases	1 / 4	2 / 24	Not quantified
Non-human reservoir hosts: Grey kangaroos, birds, or mammals	3 / 3	7 / 8	7 / 3
Elevation / Altitude	2 / 3	2 / 8	2 / 0

* Variables used as offsets or used for adjustment were not included, interactions of the variables were not included.

** N_sa_ is the number of articles that have significant exposures; N_a_ is the number of articles that used the exposures; N_sm_ is the number of models that have significant exposures; N_m_ is the number of models that used the exposures. Numbers are summarised in categories of mosquitoes and non-human reservoir hosts, which indicates one or more species are applied in each article or each model.

*** The same exposure can be applied several times with different time periods or time-lags in the same model. The associations of significant exposures and RRV infections were not quantified in some papers.

Most studies (23, 53.5% of 43 studies) used incidence rates or disease occurrence as dependent variables (Table 2). Twelve studies used outbreaks as dependent variables (Table 3), while ten used notified cases (Table 4). Two articles used incidence rates and outbreaks in different models. Linear models were applied for analysing all forms of notified data. Most studies using time-series and spatial analysis models forecast incidence rather than outbreaks. All studies using CUSUM-based models predicted outbreaks.

Only ten of the 43 studies published data related to model performance; among them, five used logistic regression models, one used a Hurdle model, one CUSUM-based methods, one a CART, one a Polynomial distributed lag model and one a negative binomial regression model (Table 6). Seven of the studies were applied to predict RRV outbreaks, one predicted incidence and two predicted cases. Most of the models achieved accuracies or overall agreements of 75.0% or higher.

**Table 6:** Model performance.

Study (author, year)	Model*	Prediction performance
Maeizer, 1999	LORM	Positive predictive value = 88%, negative predictive value = 91%.
Woodruff, 2002	LORM	Early warning models: accuracies were 64% - 100%. Late warning models: accuracies were 63% - 100%.
Gatton, 2005	LORM	Across regions, accuracies were 88%-98%, sensitivities were 0.53–0.83, and specificities were 0.94–1.00.
Hu, 2006(1)	CART	Overall agreement = 76%, Sens/Spec = 0.61/0.80.
Woodruff, 2006	LORM	Early warming model: Sens/Spec = 0.90/0.88. Late warming model: 0.85/0.98.
Pelecanos, 2010	EARS, NBC, HLM, POD and temporal analysis	True positives for four regions were 40% - 89%, 13% - 75%, 0% - 100%, 19% - 100%.
Jacups, 2011	LORM	Sensitivity/Specificity crossover were 75.8% - 88.5%.
Ng, 2014	PDL	Across regions, accuracies were 68.7% - 84.7%.
Koolhof, 2017	Hurdle model	Across regions, sensitivities were 0–1, specificities were 0.18–1.
Koolhof, 2019	NBRM	Pearson’s correlation coefficients of predicted and observed notifications ≥ 0.6 in 5 locations (5/11, 45.5%).

* LORM = Logistic Regression Model; CART = Classification and Regression Tree; EARS = Early Aberration Reporting System C1, C2 and C3 algorithms; NBC = Negative Binomial Cusum method; HLM = Historical Limits Method; POD = Poisson Outbreak Detection method; PDL = Polynomial Distributed Lag model; NBRM = Negative Binomial Regression Model.

## Discussion

This systematic review provides a complete analysis of predictive models and exposures for predicting RRV incidence. In contrast to existing reviews which described the climatic, environmental and social factors incorporated in models, this review focuses on the modelling approaches and model performance. Most predictive models used generalised linear models and time series methods, but few studies presented model performance statistics. Many exposures have been included in these models; most of them are in one or two studies only. Rainfall and temperature are the most common exposures, and within the ranges studied, the association with RRV incidence is positive for both exposures in general. Mosquito abundance has a positive effect on RRV as expected.

Data quality was assessed in few studies. This is perhaps because data were collected from government or other public data repositories; consequently, data quality is implicitly considered to be good or the quality is difficult to assess. Some models (e.g. spatial analyses) are unable to predict disease frequency and consequently model evaluation or validation approaches cannot be applied.

This systematic review identified more than 60 exposures. Climate and weather influence mosquito breeding and behaviour of hosts, and therefore change the prevalence of the disease in a complex way [64]. The lag periods for climatic exposures differ for different parts of the transmission system. Weather can accelerate or decelerate mosquito breeding over a period of several days to weeks [11, 34, 65, 66], while humans may adjust their behaviour immediately in response to weather changes, and host population structure and consequently seroprevalence may be affected by climate after a few years [10, 67, 68]. This phenomenon also explains why the same exposure can influence RRV incidence both positively and negatively at different lag times. Interactions between climatic exposures further complicate the analysis [65].

Data on vectors and reservoir host species, abundance and competence are crucial for forecasting RRV incidence [60, 69, 70]. The importance of vectors and reservoir hosts differs between species because of behavioural and ecological variation [71, 72]. The feeding and breeding of mosquito species are affected by host availability and abundance [73, 74]. Across urban, inland and coastal regions of Australia, vector and host species driving RRV transmission are diverse and variable [2]. Because of the wide variety of non-human reservoir hosts, it is extremely difficult to ascertain the complex relationships among hosts, vectors, and disease incidence. Epidemiological analyses and host ecology studies including serosurveys are important methods of detecting and describing these relationships. However, vector and reservoir host data with sufficient details and completeness to be useful for prediction are rarely available, impairing the quality of models.

Surface water sources, river flow, vegetation and remoteness, which were included only in a few studies, are promising data sources and should be explored further. Surface water and vegetation provide a favourable environment for mosquito breeding and are important for modelling [75, 76]. These exposures are increasingly incorporated in recent models [53, 61]. Inclusion of incidence terms from past weeks is also widely used in public health surveillance, e.g. the Early Aberration Reporting System, which offers aberration detection methods by analysing recent surveillance data [77].

The time-lag effects of RRV activity are generated not only by climatic factors but also by mosquito abundance, host populations and some geographical elements such as river flow and flooding [9, 15, 52, 56, 63, 78]. The time lags are also influenced by the species diversity and abundance of mosquitoes in the research area. For instance, the freshwater-breeding *Culex annulirostris* is affected by rainfall and riverine flooding at freshwater habitats, while the estuarine-breeding *Aedes vigilax* is associated with estuarine wetlands shaped by tidal flooding and rainfall [2]. Thus, analysing temporal data is helpful to identify the temporal variation in these associations with RRV incidence. Moreover, the host population, mosquito breeding and people’s lifestyle vary spatially. Data on the geographical difference and temporal trends of related exposures can be valuable for RRV prediction.

Our systematic analysis showed that linear models and time-series approaches are the two main analytical methods used to predict RRV disease. Linear regressions are simple to manipulate and explain, while time-series models are appropriate for considering autocorrelation and seasonal fluctuations. Both approaches have been widely applied in dealing with infectious diseases [79, 80]. Their pros and cons are described in some articles [81–83]. Models with good predictive performance perform well at predicting outcomes for out-of-sample data [84]. Usually cross-validation is used to assess model performance in retrospective studies, and 25% of available data for validation is recommended [84]. Some statistics can be derived, such as accuracy, specificity, sensitivity, mean-squared error, mean absolute error or root mean-squared error, for evaluation [85]. Head-to-head comparisons of models using common datasets are suggested for model assessment [86]. Robustness of the models need to be tested under various settings [86]. The best modelling approach for RRV prediction is currently unclear. Therefore, the performances of RRV predictive models are needed in order to compare them and select the best one for a given setting.

This is the first systematic review focusing on modelling approaches for predicting RRV disease. This is also the first review that lists statistical methods, significant exposures and the modelling performance of selected studies. Only studies conducted in Australia and published in English were included. We did not search grey literature. We were unable to evaluate publication bias, however two of the included studies were published without significant results. Although we have summarised broad findings in relation to exposures, we have not conducted meta-analysis. Insufficient data were available to assess performance of most models. Therefore, we were not able to strictly compare models and establish an appropriate level of confidence in their performance. The descriptions of predictive models included in this review are based on current publications, so, these data need to be interpreted with caution. The information we have presented is dependent on the limitations of the included studies. A particular issue in RRV research is the non-equivalence between routinely collected surveillance data and RRV incidence. There are also significant limitations for exposure data, brought about by site and number of weather stations, incompleteness of macropod data, variability in mosquito enumeration due to characteristics of particular trap types, and other issues.

Given the complex transmission cycle of the virus, exposures and RRV incidence would not be expected to have a simple linear relationship. Non-linear models such as generalised additive mixed models and machine learning approaches are more likely to provide a more sophisticated representation of the transmission system than linear regression [87–89]. Analytical methods that encompass climate, environmental exposures, socio-economic factors and spatio-temporal aspects for forecasting RRV incidence are also worthy of consideration. For example, Bayesian spatio-temporal modelling by Hu (2010) [45] considered the spatial effects, temporal trends, climatic exposures and an interaction term for climate exposures. Region-specific models are ideal, due to spatial variation in transmission [53]. The complex ecology and the environmental variation in Australia make it challenging to design models with universal applicability that are useful for public health programs. However, there is benefit in assessing the performance of these models, as we have done in this review, to determine usefulness, even if this means rejecting some approaches. Our work will continue with the development of RRV models for Queensland using innovative modelling approaches and then assessing their predictive performance.

Our systematic review provides an analysis of epidemiological models for predicting RRV disease using notification data in Australia. Current modelling approaches are valuable in improving understanding of RRV transmission and in predicting outbreaks. However, model performance assessments are notably lacking. Nonetheless, the summary of significant exposures provided in our systematic review offers suggestions for future modelling. Predictive models are definitely useful tools for understanding transmission and predicting outbreaks of RRV. Better data availability, combined with new modelling approaches and performance assessment may improve the accuracy of forecasting. More detailed information, like daily or weekly data on RRV cases and climatic exposures at a smaller spatial scale will improve model prediction performance [53]. RRV ecology research that provides data on the abundance or spatio-temporal distribution of the mosquitoes and non-human reservoir hosts is beneficial for modelling the transmission cycle and forecasting disease incidence.

## Supporting information

S1 Table PRISMA 2009 checklist. (DOC)

S2 Table Quality assessment criteria. (DOCX)

S3 Table Quality assessment scoring details. (DOCX)

S4 Table Details of the exposures included in models. (DOCX)

S1 Text Protocol of systematic review. (DOCX)

S2 Text Search terms. (DOCX)

## References

[1] Harley D, Sleigh A, Ritchie S. Ross river virus transmission, infection, and disease: A cross-disciplinary review. Clin Microbiol Rev. 2001;14(4):909–32. 10.1128/CMR.14.4.909-932.2001 11585790 PMC89008

[2] Claflin SB, Webb CE. Ross River Virus: Many Vectors and Unusual Hosts Make for an Unpredictable Pathogen. PLoS Path. 2015;11(9). 10.1371/journal.ppat.1005070 PMC4559463 26335937

[3] Liu X, Tharmarajah K, Taylor A. Ross River virus disease clinical presentation, pathogenesis and current therapeutic strategies. Microb Infect. 2017;19(11):496–504. 10.1016/j.micinf.2017.07.001 28754345

[4] Australia government. The National Notifiable Diseases Surveillance System. 2020 [cited 2020 17 Jan]. Available from: http://www9.health.gov.au/cda/source/cda-index.cfm

[5] Stephenson EB, Peel AJ, Reid SA, Jansen CC, McCallum H. The non-human reservoirs of Ross River virus: A systematic review of the evidence. Parasites and Vectors. 2018;11(1). 10.1186/s13071-018-2733-8 PMC5859426 29554936

[6] Dhama K, Kapoor S, Pawaiya RVS, Chakraborty S, Tiwari R, Verma AK. Ross River Virus (RRV) infection in horses and humans: a review. Pakistan Journal Of Biological Sciences: PJBS. 2014;17(6):768–79. 10.3923/pjbs.2014.768.779 26035950

[7] Tong S. Ross River virus disease in Australia: epidemiology, socioecology and public health response. Intern Med J. 2004;34(1–2):58–60. 10.1111/j.1444-0903.2004.00520.x 14748915

[8] Tong S, Dale P, Nicholls N, Mackenzie JS, Wolff R, McMichael AJ. Climate variability, social and environmental factors, and Ross River virus transmission: Research development and future research needs. Environ Health Perspect. 2008;116(12):1591–7. 10.1289/ehp.11680 19079707 PMC2599750

[9] Jacups SP, Whelan PI, Currie BJ. Ross River virus and Barmah Forest virus infections: a review of history, ecology, and predictive models, with implications for tropical northern Australia. Vector Borne And Zoonotic Diseases (Larchmont, NY). 2008;8(2):283–97. 10.1089/vbz.2007.0152 18279007

[10] Lada H, Thomson JR, Cunningham SC, Mac Nally RJAE. Rainfall in prior breeding seasons influences population size of a small marsupial. 2013;38(5):581–91. 10.1139/apnm-2012-0301 23668768

[11] Tran A, L'Ambert G, Lacour G, Benoît R, Demarchi M, Cros M, et al A rainfall-and temperature-driven abundance model for Aedes albopictus populations. 2013;10(5):1698–719. 10.3390/ijerph10051698 23624579 PMC3709343

[12] Paul M, Held L, Toschke AMJSim. Multivariate modelling of infectious disease surveillance data. 2008;27(29):6250–67. 10.1002/sim.3440 18800337

[13] Imai C, Armstrong B, Chalabi Z, Mangtani P, Hashizume MJEr. Time series regression model for infectious disease and weather. 2015;142:319–27. 10.1016/j.envres.2015.06.040 26188633

[14] Imai C, Hashizume MJTm, health. Systematic review on methodology: time series regression analysis for environmental factors and infectious diseases. 2014 10.2149/tmh.2014-21 25859149 PMC4361341

[15] Yu W, Dale P, Turner L, Tong S. Projecting the impact of climate change on the transmission of Ross River virus: methodological challenges and research needs. Epidemiol Infect. 2014;142(10):2013–23. 10.1017/S0950268814000399 24612684 PMC9151282

[16] Moher D, Liberati A, Tetzlaff J, Altman DG, The PG. Preferred Reporting Items for Systematic Reviews and Meta-Analyses: The PRISMA Statement. PLoS Med. 2009;6(7):e1000097 10.1371/journal.pmed.1000097 19621072 PMC2707599

[17] Aswi A, Cramb S, Moraga P, Mengersen KJE, Infection. Bayesian spatial and spatio-temporal approaches to modelling dengue fever: a systematic review. 2019;147. 10.1017/S0950268818002807 PMC6518570 30369335

[18] Harris RC, Sumner T, Knight GM, White RGJHv, immunotherapeutics. Systematic review of mathematical models exploring the epidemiological impact of future TB vaccines. 2016;12(11):2813–32. 10.1080/21645515.2016.1205769 27448625 PMC5137531

[19] Caro JJ, Eddy DM, Kan H, Kaltz C, Patel B, Eldessouki R, et al Questionnaire to assess relevance and credibility of modeling studies for informing health care decision making: an ISPOR-AMCP-NPC Good Practice Task Force report. 2014;17(2):174–82. 10.1016/j.jval.2014.01.003 24636375

[20] Fone D, Hollinghurst S, Temple M, Round A, Lester N, Weightman A, et al Systematic review of the use and value of computer simulation modelling in population health and health care delivery. 2003;25(4):325–35. 10.1093/pubmed/fdg075 14747592

[21] Maelzer D, Hales S, Weinstein P, Zalucki M, Woodward A. El Nino and arboviral disease prediction. Environ Health Perspect. 1999;107(10):817–8. 10.1289/ehp.99107817 10504148 PMC1566593

[22] Ryan PA, Do KA, Kay BH. Spatial and temporal analysis of Ross River virus disease patterns at Maroochy Shire, Australia: association between human morbidity and mosquito (Diptera: Culicidae) abundance. J Med Entomol. 1999;36(4):515–21. 10.1093/jmedent/36.4.515 10467782

[23] Muhar A, Dale PE, Thalib L, Arito EJEh, medicine p. The spatial distribution of Ross River virus infections in Brisbane: significance of residential location and relationships with vegetation types. 2000;4(4):184–9. 10.1007/BF02931256 21432483 PMC2723594

[24] Tong S, Hu W. Climate variation and incidence of Ross river virus in Cairns, Australia: a time-series analysis. 2001;109(12):1271–3. 10.1289/ehp.011091271 11748035 PMC1240510

[25] Done SJ, Holbrook NJ, Beggs PJ. The Quasi-Biennial Oscillation and Ross River virus incidence in Queensland, Australia. Int J Biometeorol. 2002;46(4):202–7. 10.1007/s00484-002-0137-z 12242477

[26] Tong S, Bi P, Donald K, McMichael AJ, Health C. Climate variability and Ross River virus transmission. 2002;56(8):617–21. 10.1136/jech.56.8.617 12118054 PMC1732227

[27] Tong S, Hu W. Different responses of Ross River virus to climate variability between coastline and inland cities in Queensland, Australia. Occup Environ Med. 2002;59(11):739–44. 10.1136/oem.59.11.739 12409532 PMC1740241

[28] Woodruff RE, Guest CS, Garner MG, Becker N, Lindesay J, Carvan T, et al Predicting Ross River virus epidemics from regional weather data. Epidemiology. 2002;13(4):384–93. 10.1097/00001648-200207000-00005 12094092

[29] Gatton ML, Kelly-Hope LA, Kay BH, Ryan PA. Spatial-temporal analysis of Ross River virus disease patterns in Queensland Australia. Am J Trop Med Hyg. 2004;71(5):629–35. 15569796

[30] Hu W, Nicholls N, Lindsay M, Dale P, McMichael AJ, Mackenzie JS, et al Development of a predictive model for ross river virus disease in Brisbane, Australia. Am J Trop Med Hyg. 2004;71(2):129–37. 15306700

[31] Tong S, Hu W, McMichael AJ, Health I. Climate variability and Ross River virus transmission in Townsville region, Australia, 1985–1996. 2004;9(2):298–304. 10.1046/j.1365-3156.2003.01175.x 15040569

[32] Gatton ML, Kay BH, Ryan PA. Environmental predictors of Ross River virus disease outbreaks in Queensland Australia. Am J Trop Med Hyg. 2005;72(6):792–9. 15964965

[33] Tong S, Hu W, Nicholls N, Dale P, MacKenzie J, Patz J, et al Climatic, high tide and vector variables and the transmission of Ross River virus. 2005;35(11):677–80. 10.1111/j.1445-5994.2005.00935.x 16248864

[34] Hu WB, Tong SL, Mengersen K, Oldenburg B. Rainfall, mosquito density and the transmission of Ross River virus: A time-series forecasting model. Ecol Model. 2006;196(3–4):505–14.

[35] Hu W, Tong S, Mengersen K, Oldenburg B, Dale P. Mosquito species (Diptera: Culicidae) and the transmission of Ross River virus in Brisbane, Australia. J Med Entomol. 2006;43(2):375–81. 10.1603/0022-2585(2006)043[0375:msdcat]2.0.co;2 16619624

[36] Ryan PA, Alsemgeest D, Gatton ML, Kay BH. Ross River virus disease clusters and spatial relationship with mosquito biting exposure in Redland Shire, southern Queensland, Australia. J Med Entomol. 2006;43(5):1042–59. 10.1603/0022-2585(2006)43[1042:rrvdca]2.0.co;2 17017245

[37] Woodruff RE, Guest CS, Garner MG, Becker N, Lindsay M. Early warning of Ross River virus epidemics: combining surveillance data on climate and mosquitoes. Epidemiology. 2006;17(5):569–75. 10.1097/01.ede.0000229467.92742.7b 16837824

[38] Hu W, Tong S, Mengersen K, Oldenburg B. Exploratory spatial analysis of social and environmental factors associated with the incidence of Ross River virus in Brisbane, Australia. Am J Trop Med Hyg. 2007;76(5):814–9. 17488897

[39] Jacups SP, Whelan PI, Markey PG, Cleland SJ, Williamson GJ, Currie BJ. Predictive indicators for Ross River virus infection in the Darwin area of tropical northern Australia, using long-term mosquito trapping data. Trop Med Int Health. 2008;13(7):943–52. 10.1111/j.1365-3156.2008.02095.x 18482196

[40] Jardine A, Speldewinde P, Lindsay MD, Cook A, Johansen CA, Weinstein P. Is there an association between dryland salinity and Ross River virus disease in southwestern Australia? EcoHealth. 2008;5(1):58–68. 10.1007/s10393-007-0151-z 18648798

[41] Watkins RE, Eagleson S, Veenendaal B, Wright G, Plant AJ. Applying cusum-based methods for the detection of outbreaks of Ross River virus disease in Western Australia. BMC Med Inform Decis Mak. 2008;8:37 10.1186/1472-6947-8-37 18700044 PMC2542357

[42] Barton PS, Weaver HJ. Mosquito (Diptera: Culicidae) and rainfall associations with arbovirus disease in Eastern Victoria. Trans R Soc S Aust. 2009;133(2):257–64.

[43] Bi P, Hiller JE, Cameron AS, Zhang Y, Givney R. Climate variability and Ross River virus infections in Riverland, South Australia, 1992–2004. Epidemiol Infect. 2009;137(10):1486–93. 10.1017/S0950268809002441 19296873

[44] Williams CR, Fricker SR, Kokkinn MJ. Environmental and entomological factors determining Ross River virus activity in the River Murray Valley of South Australia. Aust N Z J Public Health. 2009;33(3):284–8. 10.1111/j.1753-6405.2009.00390.x 19630851

[45] Hu W, Clements A, Williams G, Tong S, Mengersen K. Bayesian spatiotemporal analysis of socio-ecologic drivers of Ross River virus transmission in Queensland, Australia. Am J Trop Med Hyg. 2010;83(3):722–8. 10.4269/ajtmh.2010.09-0551 20810846 PMC2929077

[46] Hu W, Mengersen K, Dale P, Tong S. Difference in mosquito species (Diptera: Culicidae) and the transmission of Ross River virus between coastline and inland areas in Brisbane, Australia. Environ Entomol. 2010;39(1):88–97. 10.1603/EN07037 20146843

[47] Pelecanos AM, Ryan PA, Gatton ML. Outbreak detection algorithms for seasonal disease data: a case study using ross river virus disease. BMC Med Inf Decis Making. 2010;10(74):1–9. 10.1186/1472-6947-10-74 PMC3004813 21106104

[48] Sparks RS, Keighley T, Muscatello D. Early warning CUSUM plans for surveillance of negative binomial daily disease counts. JApS. 2010;37(11):1911.

[49] Jacups SP, Whelan PI, Harley D. Arbovirus Models to Provide Practical Management Tools for Mosquito Control and Disease Prevention in the Northern Territory, Australia. J Med Entomol. 2011;48(2):453–60. 10.1603/me10193 21485389

[50] Pelecanos AM, Ryan PA, Gatton ML. Spatial-temporal epidemiological analyses of two sympatric, co-endemic alphaviral diseases in Queensland, Australia. Vector Borne Zoonotic Dis. 2011;11(4):375–82. 10.1089/vbz.2009.0256 21466385

[51] Vally H, Peel M, Dowse GK, Cameron S, Codde JP, Hanigan I, et al Geographic information systems used to describe the link between the risk of Ross River virus infection and proximity to the Leschenault Estuary, WA. Aust N Z J Public Health. 2012;36(3):229–35. 10.1111/j.1753-6405.2012.00869.x 22672028

[52] Werner AK, Goater S, Carver S, Robertson G, Allen GR, Weinstein P. Environmental drivers of Ross River virus in southeastern Tasmania, Australia: towards strengthening public health interventions. Epidemiol Infect. 2012;140(2):359–71. 10.1017/S0950268811000446 21439102

[53] Ng V, Dear K, Harley D, McMichael A. Analysis and prediction of Ross River virus transmission in New South Wales, Australia. Vector Borne Zoonotic Dis. 2014;14(6):422–38. 10.1089/vbz.2012.1284 24745350

[54] Yu W, Mengersen K, Dale P, Mackenzie JS, Toloo GS, Wang X, et al Epidemiologic patterns of Ross River virus disease in Queensland, Australia, 2001–2011. The American Journal Of Tropical Medicine And Hygiene. 2014;91(1):109–18. 10.4269/ajtmh.13-0455 24799374 PMC4080548

[55] Rohart F, Milinovich GJ, Avril SM, Le Cao KA, Tong S, Hu W. Disease surveillance based on Internet-based linear models: an Australian case study of previously unmodeled infection diseases. Sci Rep. 2016;6:38522 10.1038/srep38522 27994231 PMC5172376

[56] Cutcher Z, Williamson E, Lynch SE, Rowe S, Clothier HJ, Firestone SM. Predictive modelling of Ross River virus notifications in southeastern Australia. Epidemiol Infect. 2017;145(3):440–50. 10.1017/S0950268816002594 27866492 PMC9507680

[57] Koolhof IS, Bettiol S, Carver S. Fine-temporal forecasting of outbreak probability and severity: Ross River virus in Western Australia. Epidemiol Infect. 2017;145(14):2949–60. 10.1017/S095026881700190X 28868994 PMC9152751

[58] Stratton MD, Ehrlich HY, Mor SM, Naumova EN. A comparative analysis of three vector-borne diseases across Australia using seasonal and meteorological models. Sci Rep. 2017;7:40186 10.1038/srep40186 28071683 PMC5223216

[59] Flies EJ, Weinstein P, Anderson SJ, Koolhof I, Foufopoulos J, Williams CR. Ross river virus and the necessity of multiscale, eco-epidemiological analyses. J Infect Dis. 2018;217(5):807–15. 10.1093/infdis/jix615 29216368

[60] Walker LJ, Selvey LA, Jardine A, Johansen CA, Lindsay MDA. Mosquito and Virus Surveillance as a Predictor of Human Ross River Virus Infection in South-West Western Australia: How Useful Is It? Am J Trop Med Hyg. 2018;99(4):1066–73. 10.4269/ajtmh.18-0459 30182918 PMC6159580

[61] Walsh MG, Webb C. Hydrological features and the ecological niches of mammalian hosts delineate elevated risk for Ross River virus epidemics in anthropogenic landscapes in Australia. Parasit Vectors. 2018;11(1):192 10.1186/s13071-018-2776-x 29554980 PMC5859420

[62] Koolhof IS, Gibney KB, Bettiol S, Charleston M, Wiethoelter A, Arnold A-L, et al The forecasting of dynamical Ross River virus outbreaks: Victoria, Australia. 2019:100377 10.1016/j.epidem.2019.100377 31735585

[63] Tall JA, Gatton ML. Flooding and Arboviral Disease: Predicting Ross River Virus Disease Outbreaks Across Inland Regions of South-Eastern Australia. J Med Entomol. 2019. 10.1093/jme/tjz120 31310648

[64] Morin CW, Semenza JC, Trtanj JM, Glass GE, Boyer C, Ebi KLJCEHR. Unexplored Opportunities: Use of Climate-and Weather-Driven Early Warning Systems to Reduce the Burden of Infectious Diseases. 2018;5(4):430–8. 10.1007/s40572-018-0221-0 30350265

[65] Costa EAPdA Santos EMdM, Correia JC Albuquerque CMRdJRBdE. Impact of small variations in temperature and humidity on the reproductive activity and survival of Aedes aegypti (Diptera, Culicidae). 2010;54(3):488–93.

[66] Abiodun GJ, Maharaj R, Witbooi P, Okosun KOJMj. Modelling the influence of temperature and rainfall on the population dynamics of Anopheles arabiensis. 2016;15(1):364. 10.1186/s12936-016-1411-6 PMC4946230 27421769

[67] Dickman CR, Haythornthwaite AS, McNaught GH, Mahon PS, Tamayo B, Letnic MJWR. Population dynamics of three species of dasyurid marsupials in arid central Australia: a 10-year study. 2001;28(5):493–506.

[68] Russell B, Smith B, Augee MJWR. Changes to a population of common ringtail possums (Pseudocheirus peregrinus) after bushfire. 2003;30(4):389–96.

[69] Flies EJ, Flies AS, Fricker SR, Weinstein P, Williams CR. Regional Comparison of Mosquito Bloodmeals in South Australia: Implications for Ross River Virus Ecology. J Med Entomol. 2016;53(4):902–10. 10.1093/jme/tjw035 27113100

[70] Koolhof IS, Carver S. Epidemic host community contribution to mosquito-borne disease transmission: Ross River virus. Epidemiol Infect. 2017;145(4):656–66. 10.1017/S0950268816002739 27890043 PMC9507737

[71] Carver S, Bestall A, Jardine A, Ostfeld RS. Influence of hosts on the ecology of arboviral transmission: potential mechanisms influencing dengue, Murray Valley encephalitis, and Ross River virus in Australia. Vector Borne Zoonotic Dis. 2009;9(1):51–64. 10.1089/vbz.2008.0040 18800866

[72] Jansen CC, Webb CE, Northill JA, Ritchie SA, Russell RC, Van Den Hurk AF. Vector Competence of Australian Mosquito Species for a North American Strain of West Nile Virus. Vector-Borne Zoonotic Dis. 2008;8(6):805–11. 10.1089/vbz.2008.0037 18973445

[73] Marm Kilpatrick A, Daszak P, Jones MJ, Marra PP, Kramer LDJPotRSBBS. Host heterogeneity dominates West Nile virus transmission. 2006;273(1599):2327–33. 10.1098/rspb.2006.3575 16928635 PMC1636093

[74] Thiemann TC, Wheeler SS, Barker CM, Reisen WKJPntd. Mosquito host selection varies seasonally with host availability and mosquito density. 2011;5(12):e1452 10.1371/journal.pntd.0001452 22206038 PMC3243726

[75] Snr S, Norma-Rashid Y, Sofian-Azirun MJWASET. Mosquitoes larval breeding habitat in urban and suburban areas, Peninsular Malaysia. 2011;58(58):569–73.

[76] Yang L, Turo KJ, Riley CB, Inocente EA, Tian J, Hoekstra NC, et al Can urban greening increase vector abundance in cities? The impact of mowing, local vegetation, and landscape composition on adult mosquito populations. 2019:1–13.

[77] Fricker RD Jr, Hegler BL, Dunfee DAJSMrn. Assessing the performance of the early aberration reporting system (EARS) Syndromic Surveillance Algorithms. 2007.

[78] McIver L, Xiao J, Lindsay MD, Rowe T, Yun G. A climate-based early warning system to predict outbreaks of Ross River virus disease in the Broome region of Western Australia. Aust N Z J Public Health. 2010;34(1):89–90. 10.1111/j.1753-6405.2010.00480.x 20920112

[79] Unkel S, Farrington CP, Garthwaite PH, Robertson C, Andrews NJJotRSSSA. Statistical methods for the prospective detection of infectious disease outbreaks: a review. 2012;175(1):49–82. 10.1016/j.jss.2011.07.032 21920558

[80] M'ikanatha NM, Lynfield R, Van Beneden CA, De Valk H. Infectious disease surveillance: Wiley Online Library; 2013.

[81] Nsoesie E, Brownstein J, Ramakrishnan N, Marathe M. A systematic review of studies on forecasting the dynamics of influenza outbreaks. Influenza Other Respir Viruses 2013. 10.1111/irv.12226 PMC4181479 24373466

[82] Naish S, Dale P, Mackenzie JS, McBride J, Mengersen K, Tong SJBid. Climate change and dengue: a critical and systematic review of quantitative modelling approaches. 2014;14(1):167. 10.1186/1471-2334-14-167 PMC3986908 24669859

[83] Racloz V, Ramsey R, Tong S, Hu WJPntd. Surveillance of dengue fever virus: a review of epidemiological models and early warning systems. 2012;6(5). 10.1371/journal.pntd.0001648 PMC3358322 22629476

[84] Giancristofaro RA, Salmaso L. Model performance analysis and model validation in logistic regression. Statistica. 2003;63(2):375–96.

[85] Unkel S, Farrington CP, Garthwaite PH, Robertson C, Andrews N. Statistical Methods for the Prospective Detection of Infectious Disease Outbreaks: A Review. J Roy Statistical Society. 2011;175(1):49–82.

[86] Chretien J-P, George D, Shaman J, Chitale RA, McKenzie FE. Influenza Forecasting in Human Populations: A Scoping Review. PLoS One. 2014;9(4):e94130 10.1371/journal.pone.0094130 24714027 PMC3979760

[87] Hu H, Wang H, Wang F, Langley D, Avram A, Liu MJSr. Prediction of influenza-like illness based on the improved artificial tree algorithm and artificial neural network. 2018;8(1):4895 10.1038/s41598-018-23075-1 29559649 PMC5861130

[88] Mathulamuthu SS, Asirvadam VS, Dass SC, Gill BS, Loshini T, editors. Predicting dengue incidences using cluster based regression on climate data 2016 6th IEEE International Conference on Control System, Computing and Engineering (ICCSCE); 2016: IEEE.

[89] Paul M, Held LJSiM. Predictive assessment of a non‐linear random effects model for multivariate time series of infectious disease counts. 2011;30(10):1118–36. 10.1002/sim.4177 21484849

