# Supplementary material for: Epidemiological models for predicting Ross River virus in Australia: A systematic review
Source: PLoS Negl Trop Dis. 2020 Sep 24;14(9):e0008621. doi: 10.1371/journal.pntd.0008621 (PMC7537878; doi:10.1371/journal.pntd.0008621)
Supplement: S2 Table — (DOCX) [file pntd.0008621.s002.docx]

**Quality assessment criteria**

| **Quality criterion** | **Scoring criteria** | **Scores** |
| --- | --- | --- |
| Objective and background |  |  |
| 1. Are the aims and objectives clarified? | 0 not stated  1 stated but vague  2 clearly stated | 0-2 |
| 2. Is the geographical area of the study clearly defined? | 0 not stated  1 stated but vague  2 clearly stated | 0-2 |
| Data |  |  |
| 3. Is the data source of Ross River virus infection clearly described? | 0 poorly described  1 some information missing  2 fully described | 0-2 |
| 4. Is the data source of covariates clearly described? | 0 poorly described  1 some information missing  2 fully described | 0-2 |
| 5. Is the quality of data considered? | 0 not stated  1 stated but vague  2 stated and considered | 0-2 |
| Model |  |  |
| 6. Is the model structure clearly described and appropriate for the research question? | 0 not appropriate model structure, or no description of model  1 incomplete description  2 complete description | 0-2 |
| 7. Are the modelling methods appropriate for the research question? | 0 not appropriate modelling method, or no description of method  1 incomplete description  2 complete description | 0-2 |
| 8. Is the model evaluated? | 0 not stated  1 evaluated but poorly described the evaluation process or presented the evaluation result  2 fully evaluated and presented | 0-2 |
| 9. Is the model validated? | 0 not stated  1 validated but poorly described the validation process or presented the validation result  2 fully validated and presented | 0-2 |
| Result |  |  |
| 10. Have the results been clearly and completely presented? | 0 not reported, very unclear  1 presented, but not fully reported  2 clearly and completely reported | 0-2 |
| 11. Are the results appropriately interpreted and discussed in context? | 0 no discussion  1 some discussion but key points and/or limitations missed  2 full discussion of key points, limitations discussed | 0-2 |
| Conflict of interest |  |  |
| 12. Is there a funding statement? | 0 No statement of funding  1 Funding stated | 0-1 |
| 13. Is there a conflict of interest statement? | 0 No statement of conflicts  1 Conflict stated | 0-1 |
| **Total score (Max 24): A > 18; B 13-18; C < 13.** | | |
