# Supplementary material for: Epidemiological models for predicting Ross River virus in Australia: A systematic review
Source: PLoS Negl Trop Dis. 2020 Sep 24;14(9):e0008621. doi: 10.1371/journal.pntd.0008621 (PMC7537878; doi:10.1371/journal.pntd.0008621)
Supplement: S3 Table — (DOCX) [file pntd.0008621.s003.docx]

**Quality assessment scoring details**

| **Study (author, year)** | **Quality criterion item** | | | | | | | | | | | | | **Notes** | **Total score^*^** |
| --- | --- | --- | --- | --- | --- | --- | --- | --- | --- | --- | --- | --- | --- | --- | --- |
|  | **1** | **2** | **3** | **4** | **5** | **6** | **7** | **8** | **9** | **10** | **11** | **12** | **13** |  |  |
| Maelzer, 1999 | 2 | 1 | 1 | 2 | 0 | 2 | 2 | 2 | 0 | 2 | 1 | 0 | 0 | Criterion 2 get 1 score for mentioned southeastern states as research areas but not indicate Tasmania; Criterion 3 get 1 score for RRV data source was obtained from literature search but not described clearly; Criterion 8 get 2 scores for the positive and negative predictive value was used for evaluation. Criterion 11 get 1 score for the results were not fully interpreted or discussed. | 15(B) |
| Ryan, 1999 | 2 | 2 | 2 | 2 | 2 | 2 | 1 | 0 | 0 | 2 | 2 | 1 | 0 | Criterion 5 get 2 scores for data standardization and transformation; Criterion 7 get 1 score for logistic regression was used for single mosquito species, which was not properly used. | 18(B) |
| Muhar, 2000 | 2 | 2 | 2 | 2 | 2 | 1 | 2 | 0 | 0 | 2 | 1 | 1 | 0 | Criterion 5 get 2 scores for correction in geocoding and data standardization; Criterion 6 get 1 score for modelling method was mentioned but not clearly described; Criterion 11 get 1 score for not fully interpreted and discussed the vegetation types with statistical significance. | 17(B) |
| Tong, 2001 | 2 | 2 | 2 | 2 | 0 | 2 | 2 | 2 | 2 | 2 | 2 | 0 | 0 |  | 20(A) |
| Done, 2002 | 2 | 2 | 2 | 2 | 0 | 1 | 2 | 1 | 0 | 1 | 2 | 0 | 0 | Criterion 6 get 1 score for modelling method was mentioned but not clearly described; Criterion 7 get 1 score for R square and Ljung-Box Q statistic was used but not stated in Methods; Criterion 10 get 1 score for the results were not fully presented. | 15(B) |
| Tong, 2002(1) | 2 | 2 | 2 | 2 | 0 | 2 | 2 | 2 | 0 | 2 | 2 | 1 | 1 |  | 20(A) |
| Tong, 2002(2) | 2 | 2 | 2 | 2 | 2 | 2 | 2 | 0 | 0 | 2 | 2 | 1 | 0 | Criterion 5 get 2 scores for missing data was considered and manipulated. | 19(A) |
| Woodruff, 2002 | 2 | 2 | 2 | 2 | 2 | 2 | 2 | 2 | 2 | 2 | 2 | 1 | 0 | Criterion 5 get 2 scores for data underestimate was considered and data interpolation method was used. | 23(A) |
| Hu, 2004 | 2 | 2 | 2 | 2 | 0 | 2 | 2 | 2 | 2 | 2 | 2 | 1 | 0 |  | 21(A) |
| Tong, 2004 | 2 | 2 | 2 | 2 | 0 | 2 | 2 | 2 | 2 | 2 | 2 | 1 | 0 |  | 21(A) |
| Gatton, 2004 | 2 | 2 | 2 | 2 | 2 | 2 | 2 | 0 | 0 | 2 | 2 | 1 | 0 | Criterion 4 get 2 scores for spatial autocorrelation was used as an covariate; Criterion 5 get 2 scores for data interpolation method was used; Criterion 6 get 2 scores for spatial and temporal modelling was used. | 19(A) |
| Gatton, 2005 | 2 | 2 | 2 | 2 | 2 | 2 | 2 | 2 | 0 | 2 | 2 | 1 | 0 | Criterion 4 get 2 scores for spatial autocorrelation was used as an covariate; Criterion 5 get 2 scores for data interpolation method was used; Criterion 6 get 2 scores for spatial and temporal modelling was used. | 21(A) |
| Tong, 2005 | 2 | 2 | 2 | 2 | 2 | 2 | 2 | 1 | 0 | 2 | 2 | 1 | 1 | Criterion 5 get 2 scores for data standardization; Criterion 8 get 1 score for the residuals were analyzed but not described clearly. | 21(A) |
| Hu, 2006(1) | 2 | 2 | 2 | 2 | 0 | 2 | 2 | 2 | 1 | 2 | 2 | 1 | 1 | Criterion 9 get 1 score for validation was mentioned but methods and results were not presented. | 21(A) |
| Hu, 2006(2) | 2 | 2 | 2 | 2 | 2 | 2 | 2 | 2 | 2 | 2 | 2 | 1 | 1 | Criterion 5 get 2 scores for missing value was considered and listwise deletion approach was used. | 24(A) |
| Woodruff, 2006 | 2 | 2 | 2 | 2 | 1 | 2 | 2 | 2 | 2 | 2 | 2 | 1 | 0 | Criterion 5 get 1 score for year-to-year differences in reporting practice was considered but not manipulated. | 22(A) |
| Ryan, 2006 | 2 | 2 | 2 | 2 | 2 | 2 | 2 | 0 | 0 | 1 | 2 | 1 | 0 | Criterion 4 get 2 scores for spatial clustering and mosquito biting complaints were used as covariates; Criterion 5 get 2 scores for missing data was considered and data standardization was used; Criterion 6 get 2 scores for spatial modelling by spatial scan statistic was used. | 18(B) |
| Hu, 2007 | 2 | 2 | 2 | 2 | 2 | 2 | 2 | 0 | 0 | 2 | 2 | 0 | 0 | Criterion 5 get 2 scores for data standardization. | 18(B) |
| Jacups, 2008 | 2 | 2 | 2 | 2 | 0 | 2 | 2 | 2 | 2 | 1 | 2 | 1 | 0 | Criterion 10 get 1 score for the p values of significant variables were not presented. | 20(A) |
| Jardine, 2008 | 2 | 2 | 2 | 2 | 0 | 2 | 2 | 2 | 0 | 2 | 2 | 1 | 0 |  | 19(A) |
| Watkins, 2008 | 2 | 2 | 2 | 2 | 1 | 2 | 2 | 2 | 0 | 2 | 2 | 1 | 1 | Criterion 4 get 2 scores for the historical Ross River virus data were used as covariates; Criterion 5 get 1 score for underestimation of Ross River virus incidence rate was mentioned, but not manipulated. | 21(A) |
| Barton, 2009 | 2 | 2 | 2 | 2 | 2 | 2 | 2 | 0 | 0 | 2 | 2 | 1 | 0 | Criterion 5 get 2 scores for data transformation was used to improve homogeneity of variance. | 19(A) |
| Williams, 2009 | 2 | 2 | 2 | 2 | 1 | 2 | 2 | 1 | 0 | 2 | 2 | 1 | 0 | Criterion 5 get 1 score for underestimation of Ross River virus infection was mentioned, but not manipulated; Criterion 8 get 1 score for R square was used but not described in Methods. | 19(A) |
| Bi, 2009 | 2 | 2 | 2 | 2 | 1 | 2 | 2 | 2 | 2 | 2 | 2 | 1 | 1 | Criterion 5 get 1 score for missing value was considered, but not manipulated. | 23(A) |
| Hu, 2010 (1) | 2 | 2 | 2 | 2 | 1 | 2 | 2 | 2 | 0 | 2 | 2 | 1 | 0 | Criterion 5 get 1 score for data interpolation was mentioned but the method was not described. | 20(A) |
| Hu, 2010 (2) | 2 | 2 | 2 | 2 | 0 | 2 | 2 | 0 | 0 | 2 | 2 | 1 | 1 |  | 18(B) |
| Pelecanos, 2010 | 2 | 2 | 2 | 2 | 0 | 2 | 2 | 2 | 0 | 2 | 2 | 1 | 1 | Criterion 4 get 2 scores for the historical Ross River virus data were used as covariates; | 20(A) |
| Sparks, 2010 | 2 | 2 | 2 | 2 | 0 | 2 | 2 | 0 | 0 | 1 | 1 | 0 | 0 | Criterion 4 get 2 scores for the historical Ross River virus data were used as covariates; Criterion 10 and 11 get 1 score for not fully presented results and not fully discussed. | 14(B) |
| Jacups, 2011 | 2 | 2 | 2 | 2 | 0 | 2 | 2 | 2 | 0 | 2 | 2 | 1 | 0 |  | 19(A) |
| Pelecanos, 2011 | 2 | 2 | 2 | 2 | 2 | 2 | 2 | 0 | 0 | 2 | 2 | 1 | 1 | Criterion 4 get 2 scores for spatial autocorrelation was used as an covariate; Criterion 5 get 2 scores for data standardization; Criterion 6 get 2 scores for spatial modelling was used. | 20(A) |
| Werner, 2012 | 2 | 2 | 2 | 2 | 2 | 2 | 2 | 0 | 0 | 2 | 2 | 0 | 1 | Criterion 5 get 2 scores for data standardization. | 19(A) |
| Vally, 2012 | 2 | 2 | 2 | 2 | 0 | 2 | 2 | 0 | 0 | 1 | 2 | 1 | 0 | Criterion 10 get 1 score for results not fully presented. | 16(B) |
| Ng, 2014 | 2 | 2 | 2 | 2 | 2 | 2 | 2 | 2 | 2 | 2 | 2 | 1 | 1 | Criterion 5 get 2 scores for data interpolation was used. | 24(A) |
| Yu, 2014 | 2 | 2 | 2 | 2 | 2 | 2 | 2 | 0 | 0 | 1 | 2 | 1 | 0 | Criterion 4 get 2 scores for spatial autocorrelation was used as an covariate; Criterion 5 get 2 scores for data standardization; Criterion 6 get 2 scores for spatial modelling was used; Criterion 10 get 1 score for results not fully presented. | 18(B) |
| Rohart, 2016 | 2 | 1 | 2 | 2 | 0 | 2 | 2 | 2 | 2 | 1 | 2 | 1 | 1 | Criterion 2 get 1 score for Australia was mentioned but not clearly defined as study area; Criterion 4 get 2 scores for terms of Google Trends was used as covariates; Criterion 10 get 1 score for results not fully presented. | 20(A) |
| Cutcher, 2017 | 2 | 2 | 2 | 2 | 0 | 2 | 2 | 2 | 2 | 2 | 2 | 1 | 1 |  | 22(A) |
| Koolhof, 2017 | 2 | 2 | 2 | 2 | 2 | 2 | 2 | 2 | 2 | 2 | 2 | 1 | 0 | Criterion 5 get 2 scores for data interpolation was used. | 23(A) |
| Stratton, 2017 | 2 | 2 | 2 | 2 | 2 | 2 | 2 | 2 | 0 | 2 | 2 | 0 | 1 | Criterion 5 get 2 scores for missing data was considered and manipulated. Criterion 10 get 2 scores for detailed results were presented in supplementary materials. | 21(A) |
| Flies, 2018 | 2 | 2 | 2 | 2 | 2 | 2 | 2 | 2 | 0 | 2 | 2 | 0 | 1 |  | 21(A) |
| Walker, 2018 | 2 | 2 | 2 | 2 | 2 | 2 | 2 | 2 | 0 | 2 | 2 | 0 | 0 | Criterion 5 get 2 scores for data exclusion was used. | 20(A) |
| Walsh, 2018 | 2 | 1 | 2 | 2 | 2 | 2 | 2 | 0 | 2 | 2 | 2 | 1 | 1 | Criterion 2 get 1 score for Australia was mentioned but not clearly defined as study area; Criterion 5 get 2 scores for data correction for potential reporting bias was used. | 21(A) |
| Tall, 2019 | 2 | 2 | 2 | 2 | 2 | 2 | 2 | 0 | 0 | 2 | 2 | 1 | 0 | Criterion 5 get 2 scores for data exclusion was used. | 19(A) |
| Koolhof, 2019 | 2 | 2 | 2 | 2 | 2 | 2 | 2 | 2 | 2 | 2 | 2 | 1 | 1 | Criterion 5 get 2 scores for weather station selection was mentioned. | 24(A) |

* Total score (Max 24): A > 18; B 13-18; C < 13.
