# Supplementary material for: Epidemiological models for predicting Ross River virus in Australia: A systematic review
Source: PLoS Negl Trop Dis. 2020 Sep 24;14(9):e0008621. doi: 10.1371/journal.pntd.0008621 (PMC7537878; doi:10.1371/journal.pntd.0008621)
Supplement: S4 Table — (DOCX) [file pntd.0008621.s004.docx]

**Details of the exposures included in models**

| **Categories of exposures** | **Exposures^*^** | **N_sa_ / N_a_^**^** | **N_sm_ / N_m_^**^** | **Association with RRV^***^** | | **Lag time** |
| --- | --- | --- | --- | --- | --- | --- |
|  |  |  |  | **Positive** | **Negative** |  |
| Mosquitoes (Vector) | Total mosquito abundance, or species undefined | 12 / 14 | 23 / 42 | 10 | 0 | 1-4 months |
|  | *Culex annulirostris* |  |  | 7 | 0 |  |
|  | *Aedes camptorhynchus* |  |  | 7 | 1 |  |
|  | *Culex australicus* |  |  | 5 | 0 |  |
|  | *Anopheles annulipes* |  |  | 3 | 1 |  |
|  | *Aedes vigilax* |  |  | 4 | 0 |  |
|  | *Culex globocoxitus* |  |  | 4 | 0 |  |
|  | *Coquillettidia linealis* |  |  | 1 | 0 |  |
|  | *Aedes funereus* |  |  | 1 | 0 |  |
|  | *Aedes notoscriptus* |  |  | 1 | 0 |  |
|  | Presence of RRV isolates in mosquitoes | 1 / 1 | 1 / 6 | 1 | 0 |  |
| Non-human reservoir hosts | Reservoir host species undefined | 3 / 3 | 7 / 8 | 0 | 0 | 1-2 years |
|  | Grey kangaroos |  |  | 3 | 3 |  |
|  | Birds |  |  | 3 | 0 |  |
|  | Mammals |  |  | 1 | 0 |  |
| Human population or cases | Human population | 0 / 3 | 0 / 5 | 0 | 0 |  |
|  | Historical RRV cases | 1 / 4 | 2 / 24 | 0 | 0 |  |
|  | Expected RRV cases | 1 / 1 | 3 / 3 | 0 | 3 |  |
| Climate | Rainfall | 20 / 25 | 45 / 69 | 47 | 14 | 1 month - 2 years |
|  | Temperature or Temperature Suitability Index | 14 / 23 | 35 / 66 | 31 | 15 | 1 month - 2 years |
|  | Tidal height or high tidal level | 10 / 15 | 19 / 45 | 17 | 6 | 2 weeks - 2 years |
|  | Humidity or relative humidity | 7 / 12 | 12 / 37 | 5 | 10 | 2 months - 2 years |
|  | Southern Oscillation Index | 4 / 9 | 6 / 26 | 5 | 1 | 2 months - 1 year |
|  | Sea Surface Temperature | 4 / 5 | 6 / 22 | 4 | 6 | 1 month - 2 years |
|  | Evaporation | 2 / 4 | 7 / 21 | 8 | 1 | 1 month - 2 years |
|  | Vapor pressure | 3 / 4 | 5 / 18 | 5 | 0 | 1 month |
|  | Stratospheric Quasi-Biennial Oscillation index | 0 / 1 | 0 / 3 | 0 | 0 |  |
|  | La Niña events | 0 / 1 | 0 / 1 | 0 | 0 |  |
| Geography and environment | River flow or river height | 5 / 5 | 5 / 19 | 5 | 2 | 1-3 months |
|  | Distance to each surface water type | 3 / 4 | 3 / 9 | 0 | 4 |  |
|  | Elevation or altitude | 2 / 3 | 2 / 8 | 2 | 0 |  |
|  | Normalized Difference Vegetation Index | 1 / 2 | 4 / 7 | 4 | 3 | 1-2 years |
|  | Vegetation | 2 / 2 | 2 / 2 | 2 | 0 |  |
|  | Flood or waterlogging | 1 / 2 | 3 / 9 | 3 | 0 | 1-3 months |
|  | Sea level pressure | 1 / 1 | 3 / 11 | 0 | 3 | 1-2 months |
|  | Water sources | 1 / 1 | 1 / 4 | 0 | 1 |  |
|  | Water-soil balance | 1 / 1 | 1 / 1 | 0 | 0 |  |
|  | Maximum Green Vegetation Fraction | 1 / 1 | 1 / 1 | 0 | 0 |  |
|  | Land salinity | 0 / 1 | 0 / 6 | 0 | 0 |  |
|  | Caravan parks | 0 / 1 | 0 / 3 | 0 | 0 |  |
|  | Sea level | 0 / 1 | 0 / 1 | 0 | 0 |  |
| Socio-economic factors | Google Trends data | 0 / 1 | 0 / 12 | 0 | 0 |  |
|  | Accessibility/Remoteness Index of Australia | 1 / 1 | 1 / 4 | 1 | 0 |  |
|  | Global human settlement "urban-ness" score | 1 / 1 | 3 / 3 | 3 | 0 |  |
|  | Index of Relative Socio-economic Disadvantage | 1 / 1 | 2 / 3 | 1 | 1 |  |
|  | Proportion of people have lower education | 1 / 1 | 1 / 1 | 1 | 0 |  |
|  | Socioeconomic Index for Areas | 0 / 1 | 0 / 1 | 0 | 0 |  |
|  | Overseas visitors | 0 / 1 | 0 / 1 | 0 | 0 |  |
|  | Indigenous population | 0 / 1 | 0 / 1 | 0 | 0 |  |
|  | Proportion of labor workers | 0 / 1 | 0 / 1 | 0 | 0 |  |
|  | Family income | 0 / 1 | 0 / 1 | 0 | 0 |  |
|  | Human migration | 0 / 1 | 0 / 1 | 0 | 0 |  |
|  | Day of the week | 0 / 1 | 0 / 1 | 0 | 0 |  |
|  | School holiday | 0 / 1 | 0 / 1 | 0 | 0 |  |
| Spatial and temporal factors | Seasonality or season | 2 / 4 | 7 / 9 | 7 | 0 |  |
|  | Spatial auto-correlation | 3 / 3 | 3 / 3 | 0 | 0 |  |
|  | Seasonal auto-regression | 2 / 2 | 2 / 3 | 0 | 2 |  |
|  | Spatial variation | 1 / 1 | 1 / 1 | 1 | 0 |  |
|  | LGA-specific temporal trends | 1 / 1 | 1 / 1 | 1 | 0 |  |
|  | Seasonal moving average | 1 / 1 | 1 / 1 | 1 | 0 |  |
|  | Auto-regression | 1 / 1 | 1 / 1 | 1 | 0 |  |
|  | Moving average | 1 / 1 | 1 / 1 | 0 | 1 |  |
|  | Intra-annual periodicity | 1 / 1 | 1 / 1 | 0 | 0 |  |
|  | Inter-annual periodicity | 0 / 1 | 0 / 1 | 0 | 0 |  |
|  | Some effects used in specific models, like seasonally oscillating temporal random effect, trend, time-lag, seasonal and transitional influences, or the annual, semi-annual and quasi-biennial cycles | 0 / - | 0 / - | 0 | 0 |  |

* Variables used as offsets or used for adjustment were not included, interactions of the variables were not included.

** N_sa_ is the number of articles that have significant exposures; N_a_ is the number of articles that used the exposures; N_sm_ is the number of models that have significant exposures; N_m_ is the number of models that used the exposures. Numbers are summarized in categories of mosquitoes and non-human reservoir hosts, which indicates one or more species are applied in each article or each model.

*** The same exposure can be applied several times with different time periods or time-lags in the same model. The associations of significant exposures and RRV infections were not quantified in some papers.
