# Supplementary material for: Epidemiological models for predicting Ross River virus in Australia: A systematic review
Source: PLoS Negl Trop Dis. 2020 Sep 24;14(9):e0008621. doi: 10.1371/journal.pntd.0008621 (PMC7537878; doi:10.1371/journal.pntd.0008621)
Supplement: S1 Text — (DOCX) [file pntd.0008621.s005.docx]

**Protocol of systematic review**

**Review title:**

Epidemiological models for predicting Ross River virus in Australia: a systematic review.

**Review team members:**

Miss Wei Qian. Mater Research Institute‐University of Queensland (MRI‐UQ)

Dr David Harley. Mater Research Institute‐University of Queensland (MRI‐UQ)

Dr Elvina Viennet. Research and Development, Australian Red Cross Lifeblood; Institute for Health and Biomedical Innovation, School of Biomedical Sciences, Queensland University of Technology (QUT)

Dr Kathryn Glass. Research School of Population Health, Australian National University

**Contact person:**

Miss Wei Qian

Email: [wei.qian@uq.net.au](mailto:wei.qian@uq.net.au)

Address: Aubigny Place, South Brisbane, Brisbane, Queensland, 4101

**Start date:**

17/06/2019

**Funding sources/sponsors:**

UQ Research Training Scholarship, Frank Clair Scholarship.

**Conflicts of interest:**

None.

**Type and method of review:**

Epidemiology, Methodology and Systematic review

**Health area of the review:**

Infections and infestations

**Review question:**

What epidemiological models have been applied to predict Ross River virus incidence or outbreaks using population-based data? What factors have these models used? How well do these models perform?

**Search databases:**

PubMed, EMBASE, Web of Science, Cochrane Library, Scopus

**Restrictions of search:**

English language, from 01 Jan 1980 to 1 Aug 2019

**Condition or domain being studied:**

Ross River virus (RRV) is the most common arboviral infection in Australia. A total of 117, 801 cases of RRV infection was reported from 1993 to 2017 in Australia. To predict the incidence of RRV, and to seek its relationship with the exposures like climate factors, geographical factors, and socio-economic factors, many models have been applied using these exposures. This systematic review aims to summarize the existing methods, their performance in predicting RRV incidence and identifying important drivers of RRV transmission.

**Participants/population:** The notified Ross River virus cases

**Intervention(s), exposure(s):** Exposures include climate factors, geographical factors, socio-economic factors, and spatial or temporal factors.

**Comparator(s)/control:** No comparator or control.

**Types of study to be included:** Observational studies.

**Context:**

Only the records that concern the Ross River virus (RRV) will be included in the systematic review. Only records used population-based data will be included. The research area should be in Australia or contain a part of Australia. Only studies used at least one epidemiological model and studied the association between factors and the RRV incidence or outbreaks will be included. Studies on genes or proteins were mainly laboratory works, which were not related to human cases of RRV infection, and therefore were excluded in this review. Studies on transfusion-related RRV transmission were excluded because the frequency is very low and we felt could safely be ignored, particularly as findings would be very difficult to integrate with studies of mosquito-transmitted disease.

**Main outcome(s):**

The performances of the models, including accuracies, sensitivities, specificities, and so on.

**Additional outcome(s):**

The associations between the factors and the Ross River virus incidence in the model including odds ratios, regression coefficients, relative risk, and so on.

**Data extraction:**

Study inclusion will be conducted by one author and checked by all the authors, and in cases of uncertainty, all authors will reach a decision after discussion. For included records, the research title, author, publication year, research area and period, predictors and predicted outcome of RRV, modelling method, significant results, prediction performance, and model validation of these studies will be extracted. Data extraction will be conducted by one author and be discussed by all authors where there are uncertainties.

**Risk of bias (quality) assessment:**

Studies will be assessed by recently published criteria for assessing observational studies. The aims and objectives, data sources, model structures, model evaluation and validation, results presentation and discussion, funding statement, and conflict of interest statement will be assessed.

**Strategy for data synthesis:**

Study characteristics and model performance will be described and tabulated. Exposures applied in these models will be summarised and their association with RRV will be listed.

**Analysis of subgroups or subsets:**

No subgroups or subsets will be analysed.

**Language:**

English

**Country:**

Australia

**Keywords:**

Ross River virus; models; exposures; prediction; incidence
