# Supplementary material for: Epidemiological models for predicting Ross River virus in Australia: A systematic review
Source: PLoS Negl Trop Dis. 2020 Sep 24;14(9):e0008621. doi: 10.1371/journal.pntd.0008621 (PMC7537878; doi:10.1371/journal.pntd.0008621)
Supplement: S2 Text — (DOCX) [file pntd.0008621.s006.docx]

**Search terms**

((((model OR models OR modelling OR modeling) in Title/Abstract/Keywords

OR

(predict OR predicting OR prediction OR predictor OR predictive) in Title/Abstract/Keywords

OR

(forecast OR forecasting) in Title/Abstract/Keywords

OR

(“early warning” OR “early warning system”) in Title/Abstract/Keywords

OR

(analysis OR analyses OR analyse OR analyze OR analysed OR analyzed OR detect OR detection) in Title/Abstract/Keywords

OR

(mathematical OR method OR methods OR statistics OR statistical OR epidemiological OR aetiological OR etiological) in Title/Abstract/Keywords

OR

(impact OR influence OR influencing OR risk OR factor OR factors) in Title/Abstract/Keywords

OR

(determinant OR determinants OR feature OR features OR parameter OR parameters OR cause OR effect) in Title/Abstract/Keywords

OR

(association OR associate OR associated OR relation OR relationship OR related) in Title/Abstract/Keywords

OR

(climate OR climatic OR environmental OR weather OR social OR economical OR season OR seasonal OR seasonality) in Title/Abstract/Keywords

OR

(spatial OR temporal OR spatial-temporal) in Title/Abstract/Keywords

OR

(mosquito OR mosquitoes OR host OR hostsl) in Title/Abstract/Keywords)

And

(“Ross River” OR “Ross River virus”) in Title/Abstract/Keywords)

NOT

(gene OR genome OR protein OR molecular OR “mouse model” OR “mice model” OR vaccine) in Title

NOT

(transfusion OR “blood donor” OR “blood donation”) in Title/Abstract/Keywords)
