# Supplementary material for: Epidemiological models for predicting Ross River virus in Australia: A systematic review
Source: PLoS Negl Trop Dis. 2020 Sep 24;14(9):e0008621. doi: 10.1371/journal.pntd.0008621 (PMC7537878; doi:10.1371/journal.pntd.0008621)
Supplement: Attachment — Submitted filename: Response to reviewers PNTD-D-20-00265.docx [file pntd.0008621.s007.docx]

**Epidemiological models for predicting Ross River virus in Australia: a systematic review**

Response to reviewers’ comments

We thank the editor and reviewers for their valuable suggestions and comments which will help us improve our manuscript. Our responses to these comments are provided below.

**Editor:**

1. Did one author review all possible articles or did multiple authors make the decisions about inclusion and assessment of the outcomes? And if multiple authors reviewed the manuscripts how was care taken to ensure they assessed the quality/inclusion in similar ways?

We thank the editor for this valuable comment. We have revised the manuscript in response (see below) and include further details here.

One author (WQ) conducted the review process and discussed with the other three authors in cases of uncertainty. All authors agreed on study selection, data extraction and study assessment after discussion and decisions were made together. All authors participated in the entire review process, provided insights independently and contributed equally to major decisions. All authors agreed on the final included articles.

The consecutive steps followed were (1) All the authors drafted the inclusion and exclusion criteria together. One author (WQ) excluded some articles by reviewing titles and abstracts, such as book chapters, then reviewed the full text of remaining papers. (2) All authors discussed and refined the inclusion and exclusion criteria in alignment with the research question. (3) Then one author (WQ) selected articles based on the refined inclusion and exclusion criteria. (4) For articles that the first author was uncertain about, such as studies that used spatial analysis or didn’t report prediction outcomes or performance, all authors reached a decision after further discussion.

The data extraction and quality assessment process were conducted in a similar way. We integrated the main review process in the discussion and used this strategy to make the process more efficient.

We added the following text to the manuscript at lines 135-136: “All the authors participated in the entire review process, discussed the main decisions and reached agreement on study selection, data extraction and study assessment together.”

2. The authors comment that meta-analysis was not possible due to a lack of similar models and model evaluations in the literature. However they do present some nice tables showing which factors predicted incidence/outbreaks/cases with Table 5 trying to summarize the top 15 predictors. In the discussion can you comment on those top 15 and expand. Some are discussed well (vectors, enviromental), but some are less well discussed (hosts).

Thank you for this comment. We have added further discussion at lines 259-262: “Because of the wide variety of non-human reservoir hosts, it is extremely difficult to ascertain the complex relationships among hosts, vectors, and disease incidence. Epidemiological analyses and host ecology studies including serosurveys are important methods of detecting and describing these relationships.”

3. In table 5 when you say "positive or negative" association with RRV can you clarify what the reference variable would be for each of the categories? i.e. is a positive association with non-human reservoirs that more reservoirs = more incidence? Etc.

Thanks for this useful comment. In all analyses, the dependent variable is RRV incidence or outbreaks. Other variables (e.g. vectors, weather) are independent.

For significant exposures in models for outbreak prediction, if the odd ratios were larger than 1 or the regression coefficients were larger than 0, this was considered to indicate a positive association with RRV disease, meaning the likelihood of RRV outbreaks increases as the exposure increases (e.g. higher vector numbers, increased rainfall).

For models predicting cases and incidence, if the coefficients, parameter estimate of the exposures or relative risk changes are larger than 0, or relative risks or incidence rate ratios are larger than 1, this is considered to indicate a positive association with RRV disease, meaning the incidence or case numbers will increase as the value of exposures get higher (e.g. higher humidity, more kangaroos).

For seasonality, whether associations are considered positive or negative depends on the definition of the exposure. In Gatton et al. (2004), in Queensland, notifications were more than twice as likely in the autumn as in the summer, with odds ratio of 2.12 (95% CI 2.06 - 2.18). In another study in South-West Western Australia (Walker et al., 2018), the authors set autumn as reference, with summer positively associated based on incidence rate ratios greater than 1.

In some articles, it was not possible to distinguish the direction of association when the statistic given was, for example, the permutation importance of exposures (Walsh and Webb, 2018) or Moran’s I statistics for evaluating the spatial autocorrelation (Gatton et al., 2004). So, exposures assessed by these statistics were not considered either as positive or negative relationships.

**Reviewer #1:**

1. The methods are clearly defined as well as the aim of the study. The study design is appropriate and with a sufficient number of studies the meet the eligible criteria defined in the methods section. There is only the need to clarify the number of researches that participated in the searches done in the different databases.

Thank you very much for these kind comments. The involvement of the four authors in the systematic review process is discussed in our response to the editor’s first comment above.

2. The conclusion is clearly stated and supported by the findings. The benefits in predicting RRV disease through the use models is stablished as well as the gaps in the use and performance assessment of the models. However, the study lacks an identification of limitations of the study and how these were addressed.

We thank the reviewer for this valuable comment. We added the following text at lines 304-311: “The descriptions of predictive models included in this review are based on current publications, so, these data need to be interpreted with caution. The information we have presented is dependent on the limitations of the included studies. A particular issue in RRV research is the non-equivalence between routinely collected surveillance data and RRV incidence. There are also significant limitations for exposure data, brought about by site and number of weather stations, incompleteness of macropod data, variability in mosquito enumeration due to characteristics of particular trap types, and other issues.”

**Reviewer #2:**

1. I appreciate that the authors made some comments at the end of their discussion on general usefulness of models and data that would be provide greater benefit for inclusion in future. There is a perennial debate among many mosquito and public health professionals on the utility of predictive models or mosquito-borne disease. It is often claimed that deficiencies in accuracy, resulting from the ecological complexity of RRV transmission cycles, mean that they are of only marginal use to local authorities in practical/operational responses. I’d like to see some additional comments from authors about the translation of this work to inform this decision making process. Are the authors recommended a particular type of model (or combination of models) as being more useful in certain circumstances? In addition, can more specific suggestions be made about the application of these models to certain geographic areas/environments or, alternatively, future modelling approaches applicable to these settings.

Thank you for these helpful comments.

We have added the following text at lines 321-327: “The complex ecology and the environmental variation in Australia make it challenging to design models with universal applicability that are useful for public health programs. However, there is benefit in assessing the performance of these models, as we have done in this review, to determine usefulness, even if this means rejecting some approaches. Our work will continue with the development of RRV models for Queensland using innovative modelling approaches and then assessing their predictive performance.”

2. The authors should also include some comments on the limitations of this work. In particular is the inconsistencies in the scope of the existing literature. While I understand that the authors are only working with published literature, there will remain some biases in that published literature that may have implications for the adaptation of results elsewhere in the country. This is especially the case where regions have not been a focus of previous modelling attempts or where some regions (e.g. NT or WA) have relatively greater focus.

We thank the reviewer for this useful comment. We added text to the discussion at lines 304-311: “The descriptions of predictive models included in this review are based on current publications, so, these data need to be interpreted with caution. The information we have presented is dependent on the limitations of the included studies. A particular issue in RRV research is the non-equivalence between routinely collected surveillance data and RRV incidence. There are also significant limitations for exposure data, brought about by site and number of weather stations, incompleteness of macropod data, variability in mosquito enumeration due to characteristics of particular trap types, and other issues.”

3. Minor comments:
     Line 61. Add “tides” to list of environmental factors
     Line 242-243. It may be worth making a note that these differences in lag between environmental/climatic factors will be influenced by differences in vector abundance/diversity within regions studied. Here, or later starting lines 265, it may be worth noting the distinct differences in key mosquitoes associated with freshwater (Culex annulirostris) and estuarine environments (Aedes vigilax).

Thanks for your suggestions. We have added “tides” into the exposure list at line 61. We added a sentence in the discussion at lines 274-278: “The time lags are also influenced by the species diversity and abundance of mosquitoes in the research area. For instance, the freshwater-breeding *Culex annulirostris* is affected by rainfall and riverine flooding at freshwater habitats, while the estuarine-breeding *Aedes vigilax* is associated with estuarine wetlands shaped by tidal flooding and rainfall. (Claflin and Webb, 2015)”

Apart from all the responses above, we updated the affiliations of one author (Elvina Viennet). We also added one acknowledgement at lines 352-354: “Australian governments fund the Australian Red Cross Lifeblood to provide blood, blood products and services to the Australian community.”

We also made some minor changes.

References:

1. Gatton ML, Kelly-Hope LA, Kay BH, Ryan PA. Spatial-temporal analysis of Ross River virus disease patterns in Queensland Australia. Am J Trop Med Hyg. 2004;71(5):629-35.

2. Walker LJ, Selvey LA, Jardine A, Johansen CA, Lindsay MDA. Mosquito and Virus Surveillance as a Predictor of Human Ross River Virus Infection in South-West Western Australia: How Useful Is It? Am J Trop Med Hyg. 2018;99(4):1066-73.

3. Walsh MG, Webb C. Hydrological features and the ecological niches of mammalian hosts delineate elevated risk for Ross River virus epidemics in anthropogenic landscapes in Australia. Parasit Vectors. 2018;11(1):192.

4. Claflin SB, Webb CE. Ross River Virus: Many Vectors and Unusual Hosts Make for an Unpredictable Pathogen. PLoS Path. 2015;11(9).
